# *Meloidogyne enterolobii*, a Major Threat to Tomato Production: Current Status and Future Prospects for Its Management

**DOI:** 10.3389/fpls.2020.606395

**Published:** 2020-11-16

**Authors:** Ashley N. Philbrick, Tika B. Adhikari, Frank J. Louws, Adrienne M. Gorny

**Affiliations:** ^1^Department of Entomology and Plant Pathology, North Carolina State University, Raleigh, NC, United States; ^2^Department of Horticultural Science, North Carolina State University, Raleigh, NC, United States

**Keywords:** root-knot nematode, population biology, disease management, RNA interference, gene editing

## Abstract

The guava root-knot nematode, *Meloidogyne enterolobii* (Syn. *M. mayaguensis*), is an emerging pathogen to many crops in the world. This nematode can cause chlorosis, stunting, and reduce yields associated with the induction of many root galls on host plants. Recently, this pathogen has been considered as a global threat for tomato (*Solanum lycopersicum* L.) production due to the lack of known resistance in commercially accepted varieties and the aggressiveness of *M. enterolobii*. Both conventional morphological and molecular approaches have been used to identify *M. enterolobii*, an important first step in an integrated management. To combat root-knot nematodes, integrated disease management strategies such as crop rotation, field sanitation, biocontrol agents, fumigants, and resistant cultivars have been developed and successfully used in the past. However, the resistance in tomato varieties mediated by known *Mi-*genes does not control *M. enterolobii*. Here, we review the current knowledge on geographic distribution, host range, population biology, control measures, and proposed future strategies to improve *M. enterolobii* control in tomato.

## Introduction

Root-knot nematodes are the most widespread soilborne plant pathogen (Agrios, 2005; Perry et al., 2009) and can cause several billion dollars of losses annually (Nicol et al., 2011; Elling, 2013). *Meloidogyne enterolobii* Yang and Eisenback, 1983, known colloquially as the guava root-knot nematode or the pacara earpod tree root-knot nematode (Yang and Eisenback, 1983), is an emerging threat due to its global distribution, wide host range, and the ability to reproduce on tomato genotypes carrying *Mi* resistance genes (Moens et al., 2009; Castagnone-Sereno, 2012). *Meloidogyne enterolobii* alone can cause up to 65% loss, which was higher than any of the other root-knot nematode species examined to date (Castagnone-Sereno, 2012; Castagnone-Sereno and Castillo, 2014). Many farmers may not even realize their fields are infected until the end of the season when crops are harvested, and they observe heavily galled root systems (Schwarz, 2019). Diagnosis of *M. enterolobii* infestation can be challenging due to morphological similarities between it and other root-knot nematode species (Blok and Powers, 2009; Castagnone-Sereno, 2012; Min et al., 2012). In the past, extensive research has been conducted under the International *Meloidogyne* Project (IMP), coordinated by leaders at North Carolina State University to assist developing countries decrease crop loss attributed to root-knot nematodes. This effort has subsequently generated identification methods and disease management strategies (Sasser et al., 1983). Recently, major research was focused on identifying new sources of genetic resistance to *M. enterolobii* due to the ability of the species to successfully reproduce on crop varieties possessing currently available resistance genes (Hunt and Handoo, 2009; Castagnone-Sereno and Castillo, 2014). Sources of potential genetic and non-host resistance have been identified in tomato (Da Silva et al., 2019), peanut, garlic, grapefruit (Rodriguez et al., 2003), guava (Castagnone-Sereno and Castillo, 2014; Chiamolera et al., 2018), plum, peach (Castagnone-Sereno and Castillo, 2014), and sweetpotato (Schwarz, 2019). Here, we review recent advances in understanding the tomato – *M. enterolobii* pathosystem conducted throughout the world. We will discuss how this progress should facilitate *M. enterolobii* management in tomato production systems. This review will also provide species information and some directions for further research on this aggressive pathogen in tomato production systems.

## Taxonomic Complexity

**FIGURE 1:**
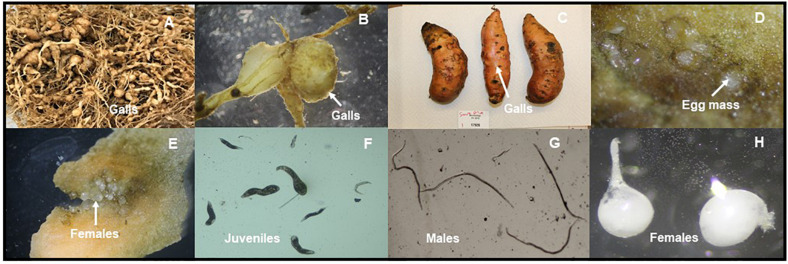
*Meloidogyne enterolobii* individuals and symptoms on different crops in North Carolina, United States (Photos provided by Dr. W. Ye). Large galls and massive root swellings of tomato cv. ‘Rutger’ in the greenhouse. The nematode was originally collected from Greene County in NC **(A)**. Galls on soybean from Johnston County, NC **(B)**. Galls on sweetpotato from Nash County, NC **(C)**. Egg masses on sweetpotato from Nash County, NC **(D)**. Adult females on sweetpotato from Nash County, NC **(E)**. Infective late second-stage juveniles (J2) from soybean in Johnston County, NC **(F)**. Males from soybean from in Wilson County, NC **(G)**. Females from sweetpotato in Johnston County, NC **(H)**.

The genus *Meloidogyne* is comprised of approximately 100 species (Hunt and Handoo, 2009; Elling, 2013; Jones et al., 2013). The name “root-knot” refers to the large galls that these nematodes induce on their hosts (Jones et al., 2013). Worldwide, there are four ‘major’ species of root-knot nematode: *M. arenaria, M. incognita, M. javanica*, and *M. hapla* (Min et al., 2012; Elling, 2013; Jones et al., 2013; Suresh et al., 2019). *M. enterolobii*, initially identified as *M. incognita*, was first discovered in the Chinese pacara earpod tree (*Enterolobium contortisiliquum*) in 1983 (Yang and Eisenback, 1983; Castagnone-Sereno, 2012). In 1988, a species identified as *Meloidogyne mayaguensis* in Puerto Rico was thought to be a new species of root-knot nematode. However, based on morphological and molecular data it was reclassified as *M. enterolobii* in 2004 (Yang and Eisenback, 1983; Castagnone-Sereno, 2012; Elling, 2013; Da Silva and Santos, 2016). The common name, guava root-knot nematode (Figure 1), was given because of the significant damage this nematode has caused to guava fruit trees (*Psidium guajava*) in South America (Carneiro et al., 2001; Schwarz et al., 2020).

## Host Range and Geographic Distribution

**FIGURE 2:**
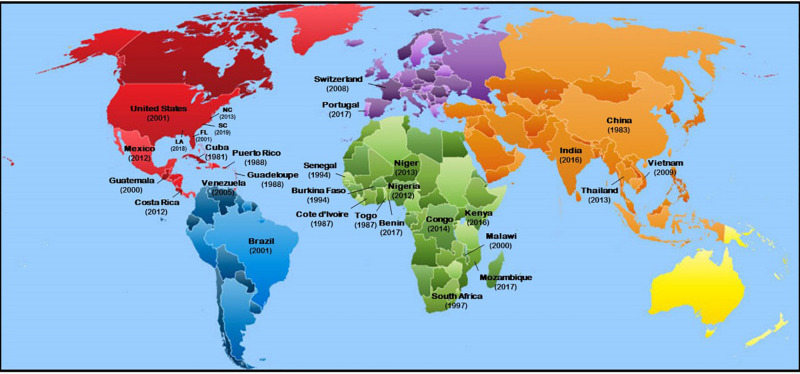
Geographic distribution of *Meloidogyne enterolobii* across the world. Numbers in parenthesis in each country indicate the year in which the nematode was reported.

*Meloidogyne enterolobii* has a broad host range (Supplementary Table S1). Only a few crop species (e.g., cabbage, corn, garlic, peanut) and several fruits (e.g., grapefruit, avocado, cashew, citrus, mango, strawberry) have been reported as non-hosts or poor hosts for *M. enterolobii* (Rodriguez et al., 2003; Brito et al., 2010; Freitas et al., 2017). This nematode has been reported worldwide (Figure 2) and established mainly in areas with a subtropical to tropical climate (Castagnone-Sereno, 2012; Elling, 2013; de Brita et al., 2018; Kirkpatrick et al., 2018; Da Silva et al., 2019; Overstreet et al., 2019; Schwarz et al., 2020). Although this nematode was originally detected in China, it has now been recorded in several African countries and South America (Elling, 2013; Da Silva et al., 2019). This nematode was also detected in commercial greenhouses in temperate regions in Switzerland (Kiewnick et al., 2009; Castagnone-Sereno and Castillo, 2014; Braun-Kiewnick et al., 2016). In the United States, *M. enterolobii* was first reported in Puerto Rico in 1988 and Florida in 2001 (Brito et al., 2004; Da Silva and Santos, 2016). It has since spread and has been reported in North and South Carolina (Rutter et al., 2019). In North Carolina, samples were originally collected and *M. enterolobii* was identified in 2011, but was not reported until 2013 (Ye et al., 2013). More recently, *M. enterolobii* was found in eight North Carolina counties: Johnston, Harnett, Sampson, Wayne, Greene, Wilson, Nash, and Columbus (Ye et al., 2013; Thiessen, 2018b; Schwarz et al., 2020). This nematode was also recently identified in sweetpotato in Louisiana.

## Biology and Life Cycle of *Meloidogyne enterolobii*

*Meloidogyne enterolobii* is an obligate biotrophic parasite and is not able to complete its life cycle without a living host (Eisenback and Triantaphyllou, 1991; Agrios, 2005; Elling, 2013). As with other root-knot nematodes, this species is an endoparasite, which feeds and matures to the adult stage of the life cycle fully inside host plant tissue (Elling, 2013; Suresh et al., 2019). *Meloidogyne enterolobii* can be distinguished from other *Meloidogyne* spp. based on the morphometrics of females, males, and juvenile stages. The most important diagnostic features are the form of a perineal pattern shape, stylet morphology of males and females, and position of the excretory pore in females; morphology of the head in the male; and the morphometrics of the head and hyaline tail in the second-stage juvenile (J2) (Yang and Eisenback, 1983).

The adult females have a white body and are pear or globe-shaped (Yang and Eisenback, 1983; Castagnone-Sereno and Castillo, 2014). Unlike adult males and J2s, the head of adult females is not distinctly set off from the neck (Yang and Eisenback, 1983; Castagnone-Sereno and Castillo, 2014). The morphometrics of *M. enterolobii* females recorded was average body length including neck 667.2 μm; body width 414.6 μm; neck length 264.8 μm; stylet length 13.4 μm; stylet knob height 2.7 μm; stylet knob width 4.3 μm; dorsal esophageal gland orifice to stylet base 3.7 μm; excretory pore not visible, and the distance from excretory pore to the head end was 178.2 μm (Yang and Eisenback, 1983; Rammah and Hirschmann, 1988). The adult males have a translucent white body and are vermiform, tapering at both ends (Yang and Eisenback, 1983; Castagnone-Sereno and Castillo, 2014). The morphometrics of males was average body length 1,496.4 μm; body width 37.0 μm; stylet length 23.6 μm; stylet knob height 2.6 μm; stylet knob width 4.6 μm; dorsal esophageal gland orifice to stylet base 4.9 μm; excretory pore to head end 165.4 μm; tail length 14.2 μm; and spicule length 28.3 μm (Yang and Eisenback, 1983; Rammah and Hirschmann, 1988). The chief features of the J2s bodies were translucent white and vermiform; truncate head region rounded; slender, and narrow tails with pointed tips, and distinct hyaline tail termini (Rammah and Hirschmann, 1988; Brito et al., 2004).

**FIGURE 3:**
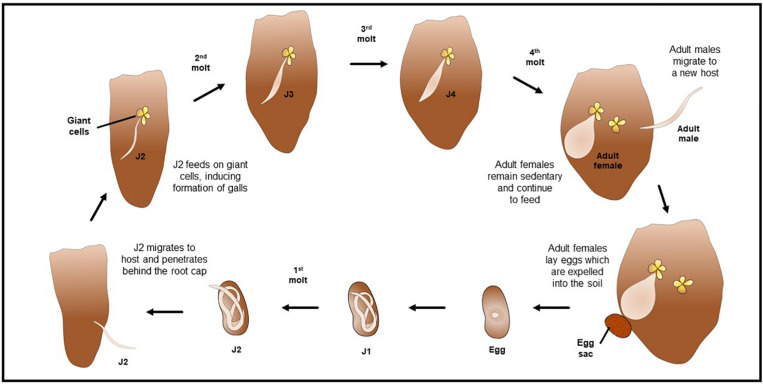
Illustration of the life cycle and root galling of *Meloidogyne enterolobii*.

Although variability in the morphometrics characters of J2 among of *M. enterolobii* isolates from different regions and countries were reported (Brito et al., 2004), the average measurements of J2s were body length 436.6 μm; body width 15.3 μm; tail length 56.4 μm; stylet length 13.0 μm; and excretory pore to head end 11.7 μm (Yang and Eisenback, 1983; Rammah and Hirschmann, 1988). Morphometrics obtained from juvenile specimens, and of the relative lengths of body, tail, and functional and replacement odontostylet (Yang and Eisenback, 1983; Rammah and Hirschmann, 1988), suggest the presence of four juvenile stages of *M. enterolobii* (Figure 3).

The life cycle of *M. enterolobii* is similar to that of other *Meloidogyne* spp. (Castagnone-Sereno and Castillo, 2014; Kirkpatrick et al., 2018; Overstreet et al., 2019). Adult females lay eggs in a protective gelatinous matrix which is usually expelled out of the root and into the soil (Moens et al., 2009; Perry and Moens, 2011; Elling, 2013; Jones et al., 2013; Castagnone-Sereno and Castillo, 2014; Overstreet et al., 2019). This matrix keeps the eggs together, protecting them from predation and extreme environmental conditions (Moens et al., 2009). After embryogenesis, the nematode develops into a first stage juvenile (J1), then undergoes a first molt to an infective J2, which hatches from the egg and is vermiform (Eisenback and Triantaphyllou, 1991; Chitwood and Perry, 2009; Moens et al., 2009; Elling, 2013; Jones et al., 2013). Hatching is dependent on both the temperature and moisture conditions of the soil (Moens et al., 2009; Perry and Moens, 2011; Elling, 2013). J2s migrate to a new host’s root system and penetrate the root epidermal tissues, usually behind the root cap (Eisenback and Triantaphyllou, 1991; Chitwood and Perry, 2009; Moens et al., 2009; Jones et al., 2013; Kirkpatrick et al., 2018; Overstreet et al., 2019). With a combination of physical damage by propelling their stylets as well as releasing cellulolytic and pectolytic enzymes, these nematodes migrate to the vascular cylinder where they establish permanent feeding sites (Eisenback and Triantaphyllou, 1991; Perry and Moens, 2011; Elling, 2013; Jones et al., 2013; Castagnone-Sereno and Castillo, 2014; Kirkpatrick et al., 2018). These feeding sites are comprised of ‘giant cells,’ which are responsible for the characteristic galls found on infected root systems (Moens et al., 2009; Elling, 2013; Jones et al., 2013; Castagnone-Sereno and Castillo, 2014; Overstreet et al., 2019). Giant cells are enlarged, multinucleated cells typically arising from plant vascular tissues that provide nutrition to nematodes by reallocating plant metabolites (Eisenback and Triantaphyllou, 1991; Moens et al., 2009; Mitchum et al., 2012). The J2 nematodes molt three more times, to the third (J3), fourth (J4) stages, and then to reproductive adults (Figure 3) (Eisenback and Triantaphyllou, 1991; Chitwood and Perry, 2009; Moens et al., 2009; Elling, 2013; Jones et al., 2013; Castagnone-Sereno and Castillo, 2014). The J3 and J4 stages do not feed as they lack a functional stylet (Chitwood and Perry, 2009; Moens et al., 2009; Jones et al., 2013).

Male *M. enterolobii* nematodes are vermiform and leave the root system, and do not feed as adults. However, males of many *Meloidogyne* spp. are only formed in unfavorable conditions, such as extreme soil temperatures, lack of sufficient soil moisture, or situations of overcrowding (Eisenback and Triantaphyllou, 1991; Chitwood and Perry, 2009; Elling, 2013; Jones et al., 2013). Females remain sedentary and continue to feed as they swell and become pear-shaped (Elling, 2013; Jones et al., 2013; Schwarz, 2019). Under favorable conditions, the life cycle of most *Meloidogyne* spp., including *M. enterolobii*, takes about 30 to 35 days to complete and each female can lay up to 600 eggs (Castagnone-Sereno and Castillo, 2014; Da Silva et al., 2019; Overstreet et al., 2019). Several generations of the life cycle may occur throughout the growing season (Chitwood and Perry, 2009).

*Meloidogyne* spp. can reproduce via amphimixis, facultative meiotic parthenogenesis, and obligate mitotic parthenogenesis (Chitwood and Perry, 2009). *Meloidogyne enterolobii* reproduces via obligate mitotic parthenogenesis or obligatory asexual reproduction (Chitwood and Perry, 2009; Castagnone-Sereno and Castillo, 2014), by which the nucleus separates into two daughter nuclei, having the same genetic material as the original (Chitwood and Perry, 2009). Males are not required for reproduction, but extreme environmental conditions may promote their development from individuals genetically disposed to become female (Eisenback and Triantaphyllou, 1991; Chitwood and Perry, 2009).

## Identification Methods and Population Biology

Traditionally, *Meloidogyne* spp. have been characterized through the morphology of adult females and males, including analysis of perineal patterns, which is the shape of cuticle folding around the vulva and anus of adult females. These methods of identification require considerable skill and experience and may otherwise lead to misidentification (Hunt and Handoo, 2009; Min et al., 2012; Elling, 2013). Some of the features of the perineal patterns were useful to separate *M. enterolobii* from other *Meloidogyne* species. In general, the perineal patterns of *M. enterolobii* are oval shape; the dorsal arch is high and round; phasmids are large, and weak lateral lines occasionally present (Karssen and van Aelst, 2001; Brito et al., 2004). However, perineal patterns within the same species may also vary in individuals from the same population, making identification difficult (Humphreys et al., 2012; Da Silva and Santos, 2016; Suresh et al., 2019). Additionally, *M. incognita* and *M. enterolobii* can have very similar perineal patterns (Humphreys et al., 2012; Schwarz, 2019; Suresh et al., 2019) and *M. enterolobii* was originally thought to be *M. incognita* based on perineal pattern analysis. The perineal pattern of *M. enterolobii* females is an oval shape, dorsal arch usually high and round, weak lateral lines sometimes present, large phasmids and has occasional breaks of striation laterally, and a circular tail tip area lacking striae (Yang and Eisenback, 1983). In addition to their perineal pattern, female root-knot nematodes can be identified to greater taxonomic groups or species by stylet morphology, body shape, or neck length. Males and J2s can be distinguished through body morphometrics or by the morphology of the head and tail (Yang and Eisenback, 1983; Eisenback and Triantaphyllou, 1991; Hunt and Handoo, 2009). However, many *Meloidogyne* species share overlapping measurements and characteristics, making discrimination at the species level difficult (Eisenback and Triantaphyllou, 1991).

Isozyme analysis is a biochemical-based diagnostic method of staining and visualizing esterase, cellulose acetate, and malate dehydrogenase (*Mdh*) isozyme profiles after separation and migration with electrophoresis (Eisenback and Triantaphyllou, 1991; Blok and Powers, 2009). Inter-species variability gives rise to these isozymes, which provide similar catalytic function but diverge in their chemical properties, such as mobility during electrophoresis (Williamson, 1991). *Meloidogyne enterolobii* can be distinguished by the unique pattern of two distinct esterase bands and one malate dehydrogenase band (Brito et al., 2004; Hunt and Handoo, 2009; Pinheiro et al., 2015; Da Silva and Santos, 2016). This method was effective to differentiate and identify young adult females to species, but not for J2s, which are most predominantly found in soil samples (Castagnone-Sereno, 2012; Elling, 2013). Also, it is highly sensitive and can be performed with extracted protein from a single adult female (Eisenback and Triantaphyllou, 1991; Brito et al., 2004; Blok and Powers, 2009). Although isozyme analysis was widely used to identify *Meloidogyne* species (Eisenback and Triantaphyllou, 1991; Blok and Powers, 2009; Hunt and Handoo, 2009; Moens et al., 2009; Castagnone-Sereno, 2012; Elling, 2013; Da Silva and Santos, 2016), this technique requires more than one polymorphic enzyme to confirm the identity of some isolates and the signal of enzyme presence or absence can vary within and across samples.

Species-specific polymerase chain reaction (PCR) assays have been developed and used to differentiate *Meloidogyne* spp. (Supplementary Table S2) (Nunn, 1992; Blok et al., 1997, 2002; Zijlstra, 2000; Long et al., 2006; Kiewnick et al., 2013). A sequence characterized amplified region (SCAR) primer set, MK7/F and MK7/R, was used to identify *M. enterolobii* (Ye et al., 2013; Villar-Luna et al., 2015; Schwarz et al., 2020). However, Schwarz et al. (2020) found that the IGS2 primers, MeF/MeR were more specific than the MK7F/MK7R primers. In another study, internal transcribed spacer (ITS) region primers, TW81F/AB28R were used to detect *M. enterolobii* (Suresh et al., 2019). Multiplex PCR has been developed to identify and detect *M. enterolobii, M. incognita*, and *M. javanica* simultaneously using DNA extracted directly from individual galls at various stages of their life cycle (Hu et al., 2011; Elling, 2013). A new quantitative real-time PCR (qPCR) assay that quantifies the amount of nucleic acid present, was developed for the specific detection, identification, and potential quantification of *M. enterolobii* in soil and plant roots (Supplementary Table S2) (Toyota et al., 2008; Kiewnick et al., 2015; Sapkota et al., 2016). Additionally, the qPCR assay showed high specificity, sensitivity, and reproducibility (Braun-Kiewnick et al., 2016). A novel satellite DNA family, pMmPet, was discovered in *M. enterolobii*, allowing species-specific identification by PCR, as well as by Southern blot and dot blot analysis (Randig et al., 2009; Elling, 2013). It was shown the satellite repeat was stable among many populations of *M. enterolobii* and high abundancy, allowing for identification of a single individual, thus making it a strong diagnostic tool (Randig et al., 2009).

Loop-mediated isothermal amplification (LAMP) technique that amplifies DNA with high specificity, sensitivity, efficiency, and rapidity under isothermal conditions has been developed (Notomi et al., 2000). Furthermore, LAMP can amplify DNA under isothermal conditions within 1 h using either two or three sets of primers. LAMP assay has been developed and used to identify *M. enterolobii, M. arenaria, M. hapla, M. incognita*, and *M. javanica* (Niu et al., 2011, 2012; Elling, 2013) and has the potential to be used as a simple screening assay in the field (Elling, 2013). High resolution melting curve (HRMC) analysis is a new, post-PCR analysis method, which is simple, fast, and use a single-tube assay method-based on PCR melting (dissociation) curve technique and can discriminate DNA sequences based on their composition, length, and GC content (Reed et al., 2007). HRMC analysis was useful to differentiate different tropical species of *Meloidogyne* (Holterman et al., 2012; Elling, 2013). HRMC technique was also applied to *M. enterolobii* in 2-step nested PCR and single-tube assay and the results showed *M. enterolobii* isolates had different melting peak patterns, with one or two peaks with different heights centered on different melting temperatures, suggesting that the risk of using a fragment that produced multiple amplicons of different length in one species (Holterman et al., 2012). However, evaluating new single copy genes and gene regions in multiplex HRMC assays might be effective to differentiate among isolates of *M. enterolobii* or *M. enterolobii* from other *Meloidogyne* spp. (Holterman et al., 2012; Elling, 2013). Analysis of single nucleotide polymorphisms (SNPs) could be a beneficial low cost and high-throughput tool for *M. enterolobii* diagnosis (Davis et al., 2005; Holterman et al., 2012). Genotyping-by-sequencing (GBS) technique discovers SNPs to whole-genome profiling of association panels (Elshire et al., 2011) and has been used successfully to investigate the phylogenetic genetic relationships of *M. enterolobii*, *M. incognita*, and *M. javanica* populations in South Africa and identify 34 SNPs that were useful to discriminate between the three *Meloidogyne* species investigated (Rashidifard et al., 2018). The complete genomes of the root-knot species *M. incognita*, *M. hapla*, and *M. enterolobii* have been sequenced and reported (Abad et al., 2008; Opperman et al., 2008; Koutsovoulos et al., 2019). Little genetic variation has been observed within the species of *M. enterolobii*, which is likely due to the mode of reproduction through mitotic parthenogenesis (Perry and Moens, 2011). DNA markers were used to test *M. enterolobii* isolates from different geographic regions and hosts and found this species was genetically homogenous (Tigano et al., 2010).

## Integrated Disease Management (IDM) Strategies

Integrated disease management (IDM) is the simultaneous use of multiple disease management strategies to suppress disease severity or incidence and reduce the pathogen population below the economic threshold level (Ciancio and Mukerji, 2007). Although IDM is an economically and ecologically sound approach, once *M. enterolobii* populations become established, the pathogen can be difficult to manage (Schwarz, 2019; Schwarz et al., 2020). Thus, identifying effective measures and integrating these into disease management plans can delay disease epidemics, reduce disease intensity, and enhance yields. Several management strategies through soil solarization, biological soil disinfestation, biological control, soil amendments, soil flooding, fumigant and non-fumigant nematicide, and host plant resistance have been employed to minimize the effects of this pathogen in crop production worldwide (Zasada et al., 2010; Noling, 2015). Further, a robust and specific diagnostic method to detect *M. enterolobii* would increase food security and improve quarantine measures to support epidemiological studies and the decision-making process of management tactics on tomato worldwide.

In the United States, particularly in North Carolina, *M. enterolobii* is under an internal quarantine, and infected material, or the nematode in any life stage, cannot be moved out of the state. *Meloidogyne enterolobii* is not transferred by tomato seed but it can be spread through sweetpotato and potato “seed” as the seed pieces (parts of the roots or tuber stems) are in contact with the soil and may become infected (Thiessen, 2018b). Thus, growers need to avoid moving infected plant material, infested soil, and contaminated farm-equipment from infested fields with *M. enterolobii* to non-infested areas (Thiessen, 2018b; Schwarz et al., 2020). However, this may be difficult to accomplish due to the high level of agricultural trade between North Carolina and the surrounding states and even international locations. It is important not to plant infected tomato transplants, but planting non-infected clean transplants is essential to avoid infesting new planting fields.

### Cultural Control

Cultural practices are non-chemical management tactics such as crop rotation with non-host crops or resistant cultivars, and these tactics are an economical method for nematode management. Crop rotation to non-host crops has a suppressive effect on *M. enterolobii* populations by inhibiting the reproduction and increase of populations through the absence of a favorable host. Rotation to non-hosts for at least 1 year can help reduce nematode populations (Schwarz, 2019). However, the rotation to non-hosts for a minimum of 3 years is recommended for tomato (Seid et al., 2015). Unfortunately, crop rotation has limits due to the broad host range of *M. enterolobii* (Thiessen, 2018a). Peanut, corn, and wheat have shown to be poor hosts for this nematode and can be utilized as rotation crops (Rodriguez et al., 2003; Brito et al., 2004; Elling, 2013; Castagnone-Sereno and Castillo, 2014; de Brita et al., 2018; Thiessen, 2018b; Schwarz et al., 2020). Weed management is another important prevention strategy because many weed species may serve as hosts to *M. enterolobii* (Rich et al., 2008; Thiessen, 2018b). Since nematodes can be easily transferred by water, farm equipment, and plant material, sanitation can prevent moving the pathogen to non-infested fields (Thiessen, 2018b). Other cultural methods such as fallowing soil, soil solarization, steaming, and flooding can be used under conducive circumstances (Seid et al., 2015; Schwarz, 2019). Additional targeted research in cultural control methods such at tillage, crop rotational plans, and soil amendments are needed to support optimal management of *M. enterolobii* in tomato. In addition, rotating tomato with non-hosts such as peanut (*Arachis hypogaea*), sour orange (*Citrus aurantium*), grapefruit (*Citrus paradise*), garlic (*Allium sativum*) (Rodriguez et al., 2003), and maize (*Zea mays*) (Guimaraes et al., 2003) could reduce *M. enterolobii* populations in soil.

### Biological Control

Biological control or biopesticide is defined as an application of live microbes (bacteria and fungi) and their gene products, essential oils, plant extracts, individual and mixed acids such as organic and amino acids, natural bioactive substances, and industrial wastes (Seid et al., 2015; Forghani and Hajihassani, 2020). Some bacterial biocontrol agents that are commercially available include *Bacillus firmus* (Bio-Nem-WP/BioSafe, Agrogreen, Ashdod, Israel), *B. firmus* GB-126 (VOTIVO^TM^, Bayer CropScience, Raleigh, NC, United States), *B. amyloliquefaciens* strain IN937a, *B. subtilis* strain GB03 (BioYield, Gustafson LLC, Plano, TX, United States), *Bacillus* spp. (Pathway Consortia, Pathway Holdings, NY, United States), and heat-killed *Burkholderia* spp. strain A396 (BioST^TM^, Albaugh, LLC, IA, United States). These biopesticides have shown a bionematicide activity against eggs, juveniles, and adults and played an important role to manage *Meloidogyne* spp. (Stirling, 2014; Seid et al., 2015; Forghani and Hajihassani, 2020). The most prominent beneficial fungi for managing *Meloidogyne* spp. are *Arthrobotrys* spp. and *Monacrosporium* spp. (Cayrol et al., 1992; Bordallo et al., 2002). These beneficial microorganisms are hematophagous fungi that use sticky mycelia to capture nematodes (Nordbring-Hertz et al., 2006). Some endophytic fungi such as *Paecilomyces* and *Trichoderma* may also trap and kill *Meloidogyne* spp. in the soil or root systems. These beneficial fungi may act at different nematode life stages such as eggs, juveniles, or adults (Schouten, 2016). *Paenibacillus* spp., is one of the growth-promoting rhizobacteria (PGPR) which strongly caused J2 mortality and reduced hatching of several *Meloidogyne* spp. including *M. enterolobii* in tomato (Bakengesa, 2016). Recently, the effects of two egg-parasitic fungi, *Pochonia chlamydosporia* and *Purpureocillium lilacinum* against *M. enterolobii* were assessed *in vitro*. Two strains CG1006 and CG1044 of *P. chlamydosporia* and CG1042 and CG1101 of *P. lilacinum* were found to be the most effective and could be potential biocontrol candidates to manage *M. enterolobii* (Forghani and Hajihassani, 2020). Thus, future research on optimizing growth conditions, efficacy and broad-spectrum action, safety, and stability of beneficial endophytic bacteria or PGPR for commercialization and utilization in IDM need to be researched for ability to control *M. enterolobii*.

Arbuscular mycorrhizal fungi (AMF) are soil fungi that form a mutualistic symbiosis with the roots of plants (Baum et al., 2015; Schouteden et al., 2015). Importantly, AMF-mediated biocontrol mechanisms include altered root morphology, enhanced plant tolerance, competition for space and nutrition with plant-parasitic nematodes, induced systemic resistance (ISR), and altered rhizosphere interactions caused by abiotic and biotic factors, including plant pathogenic nematodes (Cayrol et al., 1992; Gianinazzi et al., 2010; Smith et al., 2010; Baum et al., 2015; Schouteden et al., 2015). In the past, research has been conducted on AMF-mediated biocontrol and their potential involvement in reducing *Meloidogyne* spp. populations (Schouteden et al., 2015). With the increase in microbiome research, the development of beneficial microbial agents for field application to *M. enterolobii* is imperative in the years to come. Also, some consideration should be given toward understanding the plant root interactions with beneficial microorganisms, their symbiotic relationships, and more detailed insights into the complex mechanisms underlying biocontrol agents-mediated effects on *M. enterolobii*. Direct effects of AMF on plant-parasitic nematodes and multiple benefits (Schouteden et al., 2015) suggested that AMF could be used as a biocontrol agent for managing *M. enterolobii* and to enhance nutrient bioavailability for superior tomato fruit quality and yield.

### Chemical Control

Chemical nematicides have been used to combat *Meloidogyne* spp.; however, many of these products are being phased out due to environmental and health concerns (Elling, 2013). Two broad categories of nematicides to manage *Meloidogyne* spp., including *M. enterolobii*, are fumigants and non-fumigants. Soil fumigants are formulated as gases or liquids that quickly vaporize into gases and move through open-air spaces in the soil as a gas. Some common soil fumigants that are currently available are 1,3-dichloropropene (e.g., Telone II), metam sodium (e.g., Vapam, Sectagon-42) and metam potassium (e.g., K-Pam) (Zasada et al., 2010; Noling, 2019). Although fumigants are useful for managing *Meloidogyne* spp., they can be expensive, are subject to increased regulatory scrutiny, and do not eradicate an infested field (Zasada et al., 2010). Further, many fumigants are non-selective, also having activity on bacteria, fungi, and weed seeds in the soil. Non-fumigant nematicides are generally formulated as either granules or liquids and incorporated physically or when dissolved in water. These nematicides are either contact or systemic nematicides depending on whether they kill nematodes in soil by contact or are taken up by the plant first and then affect nematodes. Some common chemical non-fumigant nematicides used to control *Meloidogyne* spp. in the United States are fluensulfone (e.g., Nimitz, ADAMA, Raleigh, NC, United States), fluopyram (e.g., Velum Prime and Velum Total, Bayer CropScience, St. Louis, MO, United States), oxamyl (e.g., Vydate, DuPont, Wilmington, DE, United States), ethoprop (e.g., Mocap, AMVAC), and terbufos (e.g., Counter, AMVAC) (Noling, 2015; Watson and Desaeger, 2019). In the European Union and other countries in the world, fumigants metam sodium (AMVAC), and dazomet (e.g., Basamid G Certis, Columbia, MD, United States) were effective to control *M. enterolobii* populations in soil (Anonymous, 1987; Zasada et al., 2010). However, because of negative environmental side effects of these fumigants, metam sodium was recommended only be used with a minimum interval of 5 years^1^.

### Host Plant Resistance

Planting resistant varieties is the most environmentally and economically friendly method to combat root-knot nematodes in tomato (Seid et al., 2015). Plant resistance genes restrict or prevent nematode reproduction in a host. At least 10 plant resistance genes (*R*-genes; *Mi-1*, *Mi-2*, *Mi-3*, *Mi-4*, *Mi-5*, *Mi-6*, *Mi-7*, *Mi-8*, *Mi-9*, and *Mi-HT*) that confer resistance to *Meloidogyne* spp. in tomato have been identified (El-Sappah et al., 2019). Among them, only five genes (*Mi-1, Mi-3, Mi-5, Mi-9*, and *Mi-HT*) have been mapped. Compared with other *Meloidogyne* spp., *M. enterolobii* is pathogenic on crop genotypes possessing several sources of resistance genes. For example, *M. enterolobii* develops on crop genotypes carrying resistance to the major species of *Meloidogyne*, including resistant cotton, sweetpotato, tomatoes (*Mi-1* gene), potato (*Mh* gene), soybean (*Mir1* gene), bell pepper (*N* gene), sweet pepper (*Tabasco* gene) and cowpea (*Rk* gene) (Fery et al., 1998; Thies and Fery, 2000; Williamson and Roberts, 2009; Castagnone-Sereno, 2012; Quenouille et al., 2013).

The most common deployed gene, *Mi-1*, was originally identified in *Solanum peruvianum* and introgressed into *S. lycopersicum* (Da Silva et al., 2019). This gene is effective in providing resistance to *M. incognita, M. javanica*, and *M. arenaria* (Seid et al., 2015; Da Silva et al., 2019). One of the major concerns of *M. enterolobii* is that the *Mi-1* gene is not effective in controlling this species (Kiewnick et al., 2009; Seid et al., 2015; Da Silva et al., 2019). The resistance spectrum of the *Mi-1.2* gene was assessed against 15 populations of *Meloidogyne* spp. employing two contrasting tomato varieties, ‘Santa Clara’ (homozygous recessive *mi-1.2/mi-1.2*, susceptible) and ‘Debora Plus’ (heterozygous *Mi-1.2/mi-1.2*, resistant) (Gabriel et al., 2020). They found that the ‘Debora Plus’ hybrid possessing the *Mi-1.2* gene was susceptible only to *M. enterolobii* and *M. hapla* but exhibited resistance to the other 13 *Meloidogyne* spp. A great deal of effort has been put into finding new sources of resistance or tolerance to *M. enterolobii* in tomato. Da Silva et al. (2019) evaluated commercial and wild tomatoes and identified three varieties (*Solanum lycopersicum* ‘Yoshimatsu’ and ‘CNPH 1246,’ and *S. pimpinellifolium* ‘CGO 7650’ (= ‘CNPH 1195’) with tolerance to *M. enterolobii*.

Deployment of a new tomato variety by conventional breeding may take over 10 years. However, this process has been accelerated using PCR-based molecular markers linked to the *R* gene of interest, and marker-assisted selection (MAS) has been routinely used in tomato breeding programs (Foolad and Panthee, 2012; El-Sappah et al., 2019). In the absence of *M. enterolobii* resistant varieties, grafting tomatoes with resistant rootstocks could be an alternative strategy for this disease management (Louws et al., 2010; Schwarz et al., 2010; Baidya et al., 2017). Two tomato rootstocks, ‘Brigeor’ and ‘Efialto,’ showed lower reproduction for one isolate of *M. enterolobii*, but not for a second distinct isolate, indicating some differences in virulence of the isolates of *M. enterolobii* (Kiewnick et al., 2009). Yet within these breeding efforts (whether conventional or marker-assisted), special attention should be paid to genotype resistant or tolerant status to *M. enterolobii*. Plants tolerant to *M. enterolobii* are identified by minimal to no yield loss when infected, even under heavy infestation (Boerma and Hussey, 1992). However, they may still allow populations of the nematode to reproduce and increase, posing significant risk to subsequent susceptible crops and long-term *M. enterolobii* management. Although providing a robust option for avoiding short-term yield and economic losses, use of tolerant varieties should be assessed in the framework of holistic *M. enterolobii* management.

## Novel Approaches to Enhance Disease Resistance to *M. enterolobii* in Tomato

Management of *M. enterolobii* is challenging due to its broad host range, high reproductive rates, and its seemingly low economic threshold level. Recent advancements in genetic engineering have made it possible to incorporate and express indigenous and heterologous proteins from one organism to another and develop enhanced nematode resistance in plants. Strategies to engineer one or more natural resistance genes with synthetic resistance may be promising tools to suppress nematode infection and populations in tomato production systems (Gheysen et al., 1996; Jung et al., 1998; Opperman et al., 1998). However, lack of public acceptance of genetically modified organisms (GMO) tomatoes has a restricted deployment of this strategy into the market.

### Harnessing Host Plant Resistance Through Marker-Assisted Selection (MAS) in Tomato Breeding Programs

A conventional breeding program involves successive crossing and extensive phenotyping, which make this procedure labor-intensive and time-consuming. Traditionally, bi-parental mapping populations have been used to detect and identify genes or quantitative trait loci (QTL) in tomato for resistance to *Meloidogyne* spp. including *M. incognita, M. javanica, M. hapla*, and *M. enterolobii* (Kiewnick et al., 2009; Foolad and Panthee, 2012; El-Sappah et al., 2019). Genome-wide association studies (GWAS) is a powerful technique to identify SNP markers associated with QTL in cultivated and wild tomato (Hirakawa et al., 2013). The integration of biotechnology techniques into a breeding program can greatly reduce this time to incorporate new resistance genes. Genomics-assisted breeding contributes to advance MAS for evaluating tomato germplasm collections, characterizing populations, finding markers linked to specific alleles of important genes, and stacking disease resistance genes for multiple pathogens including for root-knot nematode management (Arens et al., 2010). For example, the *Mi* region contains two *Mi1-1* and *Mi1-2* genes (Milligan et al., 1998). The *Mi1-2* gene, but not *Mi1-1*, has been suggested to confer resistance to *M. incognita, M. javanica*, and *M. arenaria* (Milligan et al., 1998). The PCR markers tightly linked to *Mi1-2* (Goggin et al., 2004) and *Mi-3* (Yaghoobi et al., 2005) were reported; however, the practical use of these resistance loci (*Mi-2* to *Mi-9*) has not yet been investigated thoroughly. These resistance genes should be assessed to different isolates of *M. enterolobii* and molecular markers linked to these *Mi*-genes as well as other disease resistance genes that are needed to evaluate for their stability in tomato (Arens et al., 2010). Recent advances in whole-genome sequencing have identified large numbers of SNPs and can facilitate the use of MAS more effectively in breeding programs. KASP (Kompetitive Allele Specific PCR), is a novel competitive allele specific PCR for SNP genotyping assay based on dual FRET (Fluorescent Resonance Energy Transfer) (Broccanello et al., 2018). Recently, sequences of SNP markers for the *Mi-1* gene for resistance to root-knot nematodes have been converted using KASP assay and used in tomato breeding (Devran et al., 2016). KASP assays are less expensive, highly reproducible, and flexible compared to other marker systems (Semagn et al., 2014). Thus, characterization of a large set of tomato varieties with SNP markers would be useful for the identification of markers linked to genes for resistance to *M. enterolobii* in tomato. Also, SNPs can be converted to KASP markers and used for the MAS gene pyramiding (Devran et al., 2016).

### Developing Transgenic Plants Harboring Previously Cloned Resistance Genes

Genetic engineering offers an alternative to conventional breeding and is mainly focused on two strategies: (i) the transfer of the cloned resistance gene from other plants to tomato, and (ii) the transfer of the *Mi* resistance gene from resistant varieties to susceptible ones with highly desirable production qualities (El-Sappah et al., 2019). The *Ma* locus, which has been mapped to chromosome 7 of Myrobalan plum (*Prunus cerasifera*), has been cloned by a positional cloning approach (Claverie et al., 2011; Khallouk et al., 2011). The subsequent *Agrobacterium rhizogenes*-mediated hairy-root transgenic *Prunus* plants corroborated that the *Ma* locus conferred resistance to *M. arenaria, M. incognita*, *M. javanica, M. floridensis*, and *M. enterolobii* (Bosselut et al., 2011; Claverie et al., 2011). The *Ma* toll/interleukin-1 receptor-like nucleotide binding-leucine-rich repeat (TNL) gene confers high-level and wide-spectrum resistance to *M. incognita*, *M. arenaria*, and *M. javanica* and *M. enterolobii*, and TNL is possibly a candidate gene for the *Ma* locus (Bosselut et al., 2011; Claverie et al., 2011). Furthermore, the *Ma* – *M. enterolobii* interaction may provide a great opportunity to decipher nematode effector recognition and TNL signaling (Claverie et al., 2011).

Proteinase inhibitors (PIs) are protein molecules secreted by pathogens, which inhibit the function of proteinases and proteases released by the pathogens (Ali et al., 2017). In *Meloidogyne* spp., PIs become active against all the four classes of proteinases from nematodes such as serine, cysteine, metalloproteinases, and aspartic. Transgenic expression of PIs is a method for managing *Meloidogyne* spp. (Hepher and Atkinson, 1992; Ali et al., 2017). For example, a modified rice cystatin gene (a cysteine proteinase inhibitor) in transgenic *Arabidopsis*, reduced nematode feeding, and fecundity of *M. incognita* females (Urwin et al., 1997). The pyramiding expression system produced synergistic effects by utilizing the two defense responsive genes: a plant *cysteine proteinase inhibitor* (*CeCPI*) and a fungal *chitinase* (*PjCHI-1*) in transgenic tomato and protected all growth stages of *M. incognita* infections (Chan et al., 2015). Future research to investigate interactions between these proteinases and *M. enterolobii* could be a novel approach to manage this nematode in tomato. However, concerns about the durability of such a transgenic resistance and the consumer’s acceptance of transgenic tomato will need to be investigated.

### Utilizing Host Generated RNA Interference (RNAi) to Silence Nematode Specific Effector Genes

RNA interference (RNAi) has emerged as a powerful strategy to downregulate gene activity and has also proven effective as a control tactic against *Meloidogyne* spp. (Elling, 2013). First described for *Caenorhabditis elegans*, RNAi has been used for silencing genes by suppressing their expression in a wide variety of organisms including plant-parasitic nematodes (Huang et al., 2006b; Ali et al., 2017). In this novel strategy, genes expressed in a range of cell types are silenced when nematodes take up double-stranded RNA (dsRNA) or short interfering RNAs (siRNAs) that elicit a systemic RNAi response (Lilley et al., 2012). These dsRNA molecules ranged from 42 to 1300 bp and were effective in inducing RNAi in both cyst and root-knot nematodes (Lilley et al., 2012). *Meloidogyne* spp. synthesizes effector proteins encoded by parasitism genes, and these effectors represent the molecular interface between the nematode and host (Elling, 2013). The nematode-secreted effectors produced within the esophageal glands play critical roles in parasitism (Davis et al., 2004; Baum et al., 2007; Haegeman et al., 2012). Such developments need to be coupled with an investigation of the mechanisms by which nematodes circumvent resistance (Williamson and Kumar, 2006). The feasibility of silencing nematode genes in the host plants using RNAi has been demonstrated in *Meloidogyne* spp. (Huang et al., 2006a; Yadav et al., 2006). For example, a secreted parasitism protein called 16D10, which is expressed in the subventral esophageal gland cells of multiple *Meloidogyne* spp. and interact directly with a host intracellular transcription regulator (Huang et al., 2006b). Furthermore, the silencing of the *16D10* gene by expressing dsRNA in transgenic *Arabidopsis* enabled the development of transgenic plants that were constitutively resistant to *M. arenaria*, *M. hapla*, *M. incognita*, and *M. javanica* (Huang et al., 2006a, b).

The translationally controlled tumor protein (TCTP) was first identified in mice (Yenofsky et al., 1982). A novel *M. enterolobii* TCTP effector, named *MeTCTP* was able to promote parasitism, probably by suppressing programmed cell death in the host (Zhuo et al., 2017). The silencing of the effector *MeTCTP* resulted in a reduction in parasitism and reproductive potential of *M. enterolobii*, providing evidence of the nematode effector gene as a target for host generated RNAi to achieve disease resistance (Zhuo et al., 2017). Recently, both genome sequence data and new bioinformatics tools have emerged for developing effective dsRNA constructs and stacking of dsRNA sequences to target multiple genes for nematode control (Banerjee et al., 2017). Identification and functional analysis of nematode effector targets using RNAi technology may hold great promise for enhancing plant resistance to *M. enterolobii* in tomato.

### Exploiting Efficient Genome Editing Using the CRISPR-Cas9 Technique

The development of the clustered regularly interspaced short palindromic repeats (CRISPR) technology has become a powerful alternative to RNAi for gene silencing (Ali et al., 2019). The CRISPR/Cas9 technique incorporates foreign DNA sequences into host CRISPR loci to generate short CRISPR RNAs (crRNAs) that direct sequence-specific cleavage of homologous target double-stranded DNA by Cas endonucleases (Jinek et al., 2012). The CRISPR-Cas9 genome editing protocols have been established in the free-living nematode, *Caenorhabditis elegans* (Friedland et al., 2013; Dickinson and Goldstein, 2016) which creates DNA modification at specific loci and selects the T-DNA-free mutant (Banerjee et al., 2017). The recent availability of genome sequences for tomato (Sato et al., 2012) and *M. enterolobii* (Szitenberg et al., 2017; Koutsovoulos et al., 2019) could lead to the identification of both host and pathogen novel genes involved in the infection stage and help develop the CRISPR-Cas9 technique for enhancing the resistance to *M. enterolobii* in tomato.

## Conclusion and Perspectives

We have highlighted the progress made by several research groups in the biology and management of *M. enterolobii* in tomato using both conventional and modern technologies. Even with successes in managing other *Meloidogyne* spp. through host resistance, cultural, chemical, and biological control, the recent identification of highly virulent and aggressive nematode, *M. enterolobii*, poses a threat to tomato production globally. To manage this emerging pathogen, substantial investments are necessary to lead fundamental research focused on assessing the pathogen virulence and understanding the species identity, genetic diversity, population genetic structure, evolution, and parasitism mechanisms at a more detailed scale. Whole-genome sequences of *Meloidogyne* spp. will provide opportunities to identify the widespread occurrence of horizontally transferred genes encoding for unique effectors, contributing to successful plant parasitism in nematodes and in the modulation of the plant’s defense system, the establishment of a nematode feeding site, and the synthesis or processing of nutrients (Haegeman et al., 2011). Comparative genomic analyses across *Meloidogyne* spp. need to be exploited to advance understanding of the evolutionary relationships and population genetic structure of *M. enterolobii*. More importantly, the development of robust and specific diagnostic molecular markers is necessary to correctly identify *M. enterolobii* and prevent further spread of this highly destructive nematode. To ensure global food security, modern technologies in conjunction with classical methods should be a key priority for income generation, and sustainability to tomato growers and stakeholders (Barker, 2003). New insights into the current and future risks, supported by a more robust understanding of the interactions between tomato and *M. enterolobii* will enhance the opportunities for developing novel management tools as the ability to use chemical pesticides decrease and the need for food production continues to increase. Strengthening research collaborations and combining multidisciplinary experts working on *M. enterolobii* is required to combat this economically devastating nematode in tomato production systems.

## Author Contributions

TA and FL discussed and conceived ideas. TA designed the scope of the study. AP and TA wrote the manuscript. AG helped to revise the manuscript and offer additional discussion. All authors have read, edited, and approved it for publication.

## Conflict of Interest

The authors declare that the research was conducted in the absence of any commercial or financial relationships that could be construed as a potential conflict of interest.

## Footnotes

1 http://www.ctb.agro.nl
