# Supplementary material for: Meloidogyne enterolobii, a Major Threat to Tomato Production: Current Status and Future Prospects for Its Management
Source: Front Plant Sci. 2020 Nov 16;11:606395. doi: 10.3389/fpls.2020.606395 (PMC7701057; doi:10.3389/fpls.2020.606395)
Supplement: Supplementary file 1 [file Table_1.DOCX]

**Supplementary Table 1.** Host ranges of Meloidogyne enterolobii reported worldwide (Rodriguez et al., 2003; Brito et al., 2010,

Freitas et al., 2017).

| **Common name** | **Botanical name** | **Family** |
| --- | --- | --- |
| Arrow root | *Maranta arundinacea* | Marantaceae |
| Aubergine | *Solanum melongena* | Solanaceae |
| Banana | *Musa* spp. | Musaceae |
| Barbados cherry | *Malpighia glabra* | Malpighiaceae |
| Bell pepper | *Capsicum annuum* | Solanaceae |
| Black mulberry | *Morus nigra* | Moraceae |
| Bottle brush | *Callistemon citrinus* | Myrtaceae |
| Cape gooseberry | *Physalis peruviana* | Solanaceae |
| Carrot | *Daucus carota* | Apiaceae |
| Cassava | *Manihot esculenta* | Euphorbiaceae |
| Chinese date | *Ziziphus jujuba* | Rhamnaceae |
| Coffee | *Coffea arabica* | Rubiaceae |
| Common bean | *Phaseolus vulgaris* | Fabaceae |
| Cotton | *Gossypium hirsutum* | Malvaceae |
| Cucumber | *Cucumis sativus* | Cucurbitaceae |
| Guava | *Psidium guajava* | Myrtaceae |
| Ginger | *Zingiber officinale* | Zingiberaceae |
| Hedge cactus | *Cereus hildmannianus* | Cactaceae |
| Jackfruit | *Artocarpus heterophyllus* | Moraceae |
| Jamaican poinsettia | *Euphorbia punicea* | Euphorbiaceae |
| Japanese blueberry | *Elaeocarpus decipiens* | Elaeocarpaceae |
| Jerusalem cherry | *Solanum pseudocapsicum* | Solanaceae |
| Mulberry | *Morus alba* | Moraceae |
| Nanche | *Byrsonima cydoniifolia* | Malpighiaceae |
| Naranjilla | *Solanum quitoense* | Solanaceae |
| Papaya | *Carica papaya* | Caricaceae |
| Potato | *Solanum tuberosum* | Solanaceae |
| Soybean | *Glycine max* | Fabaceae |
| Sweet potato | *Ipomoea batatas* | Convolvulaceae |
| Tamboril | *Enterolobium contortisiliquum* | Fabaceae |
| Tobacco | *Nicotiana tabacum* | Solanaceae |
| Tomato | *Solanum lycopersicum* | Solanaceae |
| Watermelon | *Citrullus lanatus* | Cucurbitaceae |
| Yam | *Dioscorea rotundata* | Dioscoreaceae |
|  |  |  |

**Supplementary Table 2.** Diagnostic methods for the identification of Meloidogyne enterolobii infecting field crops.

| **Identifi**  **-cation method** | **Primer code** | **Primer sequence (5’-3’)** | **Ampli-con size (bp)** | **References** | **Advantages** | **Disadvantages** |
| --- | --- | --- | --- | --- | --- | --- |
| Morphology  (perineal patterns) |  |  |  | Yang and Eisenback, (1983); Eisenback and Triantaphyllou, (1991); Hunt and Handoo (2009) | The traditional method of identification.  A lot of data present on *Meloidogyne* morphology. | Requires experienced nematologists.  Can lead to misidentification. |
| Isozyme analysis |  |  |  | Eisenback and Triantaphyllou (1991); Moens et al. (2009) | Many *Meloidogyne* spp. have known, unique esterase and malate dehydrogenase patterns.  Can use single adult female. | Limited to adult females. |
| Polymerase chain reaction (PCR) with universal primers | Universal primers (5S – 18S rDNA)  Universal primers (D2/D3 28S rDNA) | TTAACTTGCCAGATCGGACG /TCTAATGAGCCGTACGC  ACAAGTACCGTGAGGGAAAGT/  TGCGAAGGAACCAGCTACTA | 720  500 | Blok et al. (1997)  Nunn (1992) | Useful for phylogenetic and diversity studies  More effective for taxonomic resolution at the genus level. | Less sequence reliability.  Identification at lower taxonomic levels and classification accuracy is reduced due to nonspecific amplification among nontarget nematode species. |
| Polymerase chain reaction (PCR) with sequence characterized amplified region (SCAR) | SCAR primers  SCAR primers (MK7-F/R  SCAR primers (Me-F/ R)  SCAR primers  (Mi2F4/ Mi1R)  SCAR primers  (Fjav/Rjav) | GAAATTGCTTTATTGTTACTAAG/ TAGCCACAGCAAAATAGTTTTC  GATCAGAGGCGGGCGCATTGCGA/CGAACTCGCTCGAACTCGAC  AACTTTTGTGAAAGTGCCGCTG / TCAGTTCAGGCAGGATCAACC  ATGAAGCTAAGACTTTGGGCT/ TCCCGCTACACCCTCAACTTC    GGTGCGCGATTGAACTGAGC/ CAGGCCCTTCAGTGGAACTATAC | 322  520  236  300  670 | Blok et al. (2002)  Tigano et al. (2010)  Long et al. (2006)  Kiewnick et al. (2013)  Zijlstra (2000) | Many species-specific primers are available for *Meloidogyne* spp.  Simpler patterns than RAPDs (locus-specific) and codominant markers.  Highly sensitive and robust assay.  Labor non-intensive.  Preformed with any life stage. | Requires expensive equipment and expense in designing specific primers.  Sequence information of primers. |
| SYBR Green quantitative real-time PCR | RKNF/ RKNR | GCTGGTGTCTAAGTGTTGCTGATAC/GAGCCTAGTGATCCACCGATAAG |  | Toyota et al. (2008) | Monitor the amplification of any double-stranded DNA sequence.  No probe is required, which can reduce assay setup and running costs. | May generate false positive signals because the SYBR dye binds to any double-stranded DNA, and it can also bind to nonspecific double-stranded DNA sequences. |
| TaqMan quantitative  real-time PCR | Ment17F/R  Probe | TGTGGTGGCTCATTTTCATTA/  AAAAACCCTAAAAATACCCCAAA  AGGAGCTG |  | Kiewnick et al. (2015) | Specific hybridization between probe and target is required to generate a fluorescent signal.  Probes can be labeled with different dyes, which allows amplification and detection of two distinct sequences in one reaction. | The synthesis of different probes is required for different sequences.  More expensive than SYBR Green assay. |
|  | F/R  Probe | TGGTTCAGGGTCATTTTTCTATAAAGT/ CAAATCGCTGCGTACCAACA  FAM-CCATTGGCACTATAAC-MGB |  | Sapkota et al. (2016) | Detect and identify target nematode DNA in complex  DNA backgrounds. |  |
| Loop-mediated isothermal amplification (LAMP) | Me-F3/ B3  Me-FIP/BIP  Me-LF/LB | CCAAGTACTAAGGAAGCCC/  ATCCTAATTTTYCTCCCACACA  ACAGTGATTACGACCATACCGCGTTCGTTGCTTAACTTGCCAGA/ TCTAAGGCAAAGTGGGCGGAGCTCTYTTTGCCTTAAACCATTCC  AAGCACGCCATCCCGTC/  TGTTGTTCGCTGTTCGC |  | Niu et al. (2011)  Niu et al. (2012)  Niu et al. (2012) | Could be detected by a cheap turbidity-meter and thus, does not require expensive equipment.  High specificity and, sensitivity (up to 10 times more sensitive  than PCR) under isothermal conditions using a set of four specially designed primers.  Can be done quickly, in a field setting. | The usefulness of LAMP methods can be limited by the presence of inhibitors in the sample.  Primer design is complex.  Unable to perform multiplex amplification. |
| Whole-genome sequencing |  | *Meloidogyne incognita*  Meloidogyne enterolobii  *Meloidogyne hapla* |  | Abad et al. (2008)  Koutsovoulos et al. (2019)  Opperman et al. (2008) | Technical accuracy and reliable.  Scientific information with genetic variants and potential values with future implications | Requires knowledge of the genome and complex bioinformatic tools  Structural variants.  Lack of laboratory validity. |
