# Supplementary figures and images for: Meloidogyne enterolobii, a Major Threat to Tomato Production: Current Status and Future Prospects for Its Management
Source: Front Plant Sci. 2020 Nov 16;11:606395. doi: 10.3389/fpls.2020.606395 (PMC7701057; doi:10.3389/fpls.2020.606395)

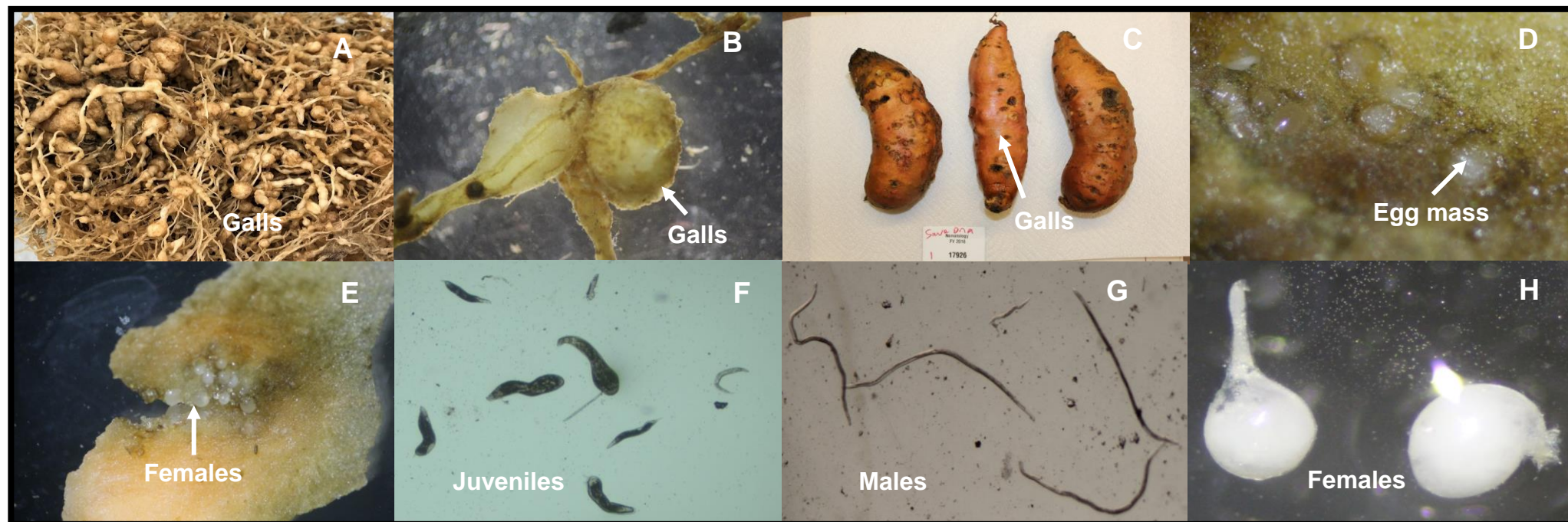

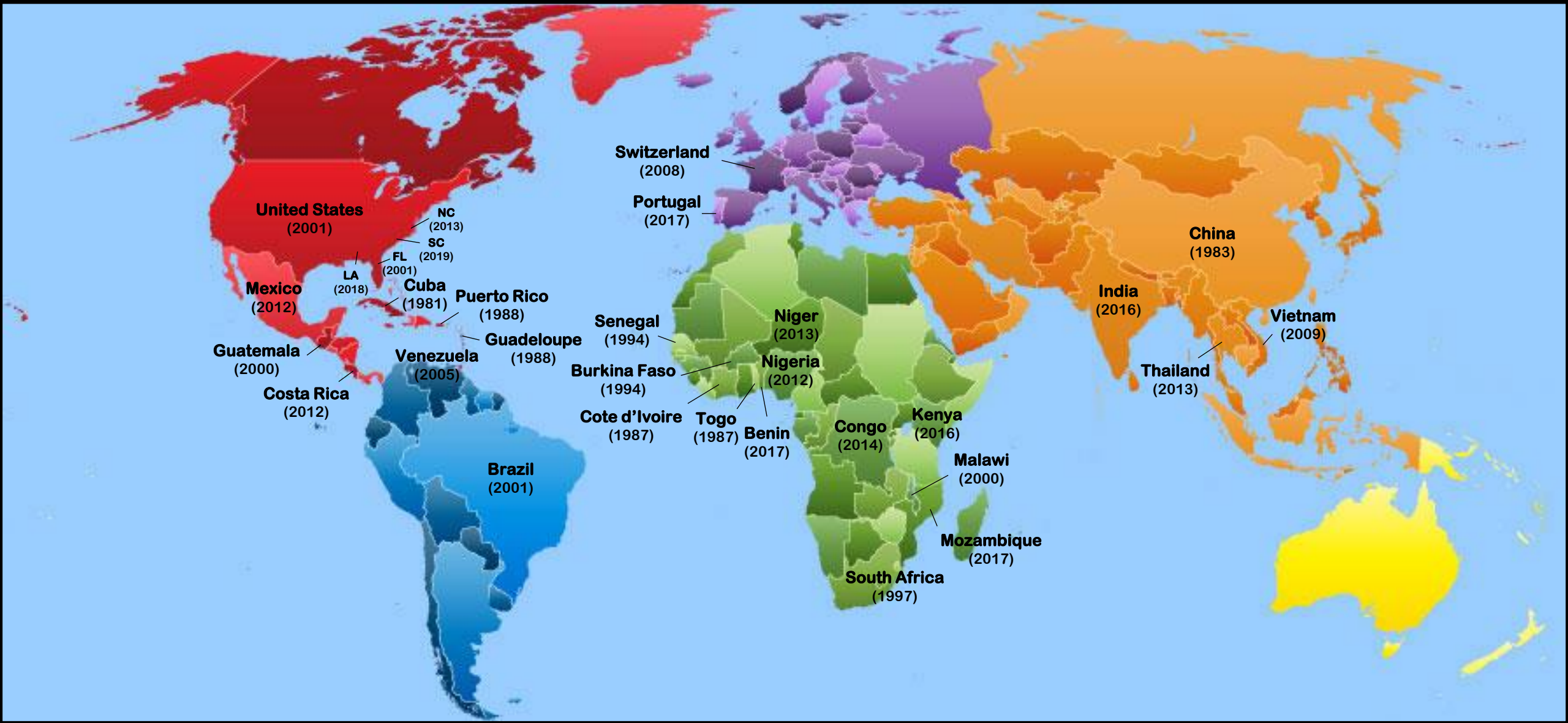

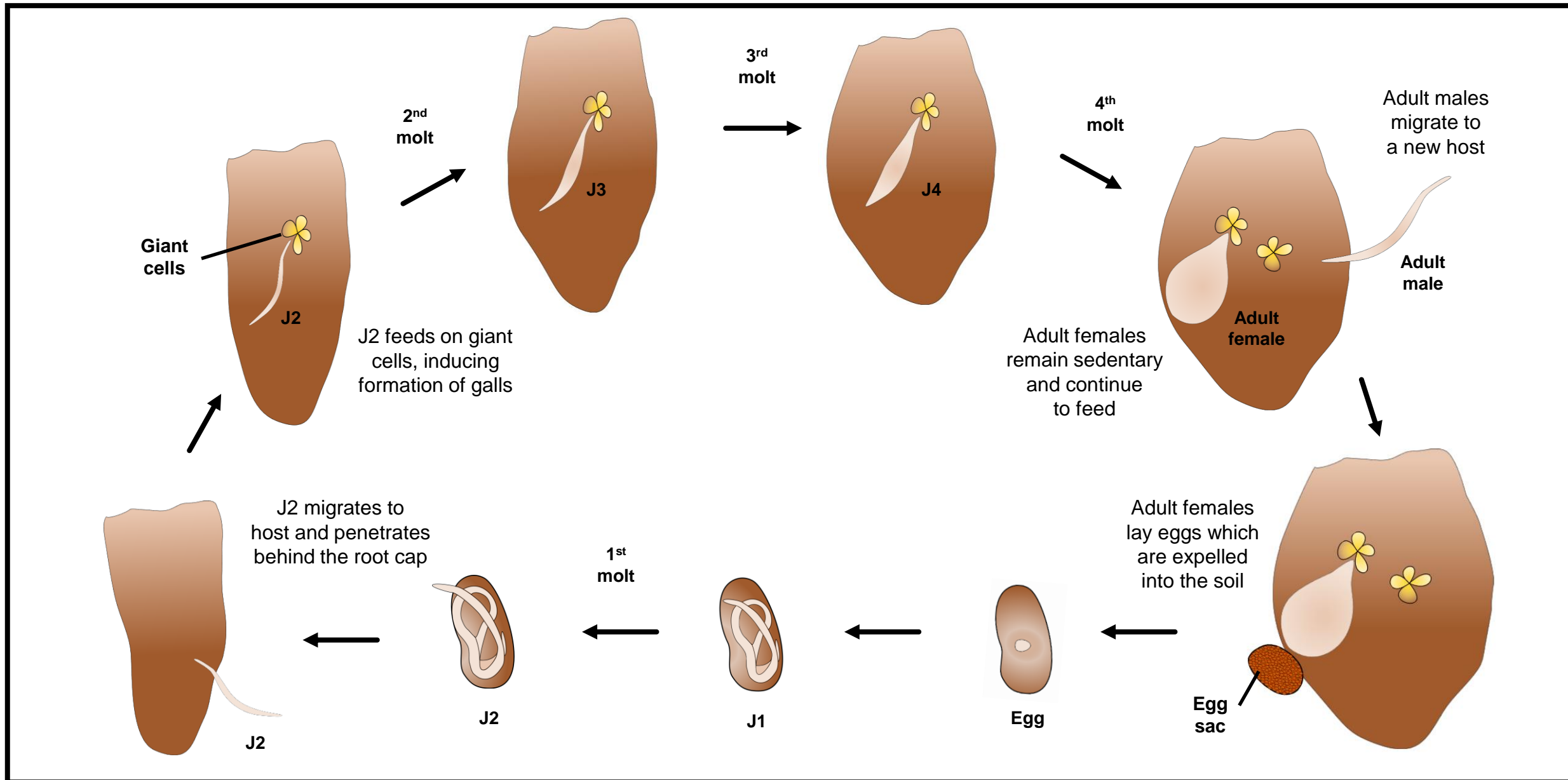

Supplement: Supplementary file 2 [file Data_Sheet_2.PDF]
